# Miscellaneous Notes

**Published:** 1872-07

**Authors:** 


### MISCELLANEOUS NOTES.

Sponge Tents as a Means for Spontaneous Expulsion of Placenta Retained in toto after Abortion.—Dr. Kreis publishes (*Berliner Klinische Wochenschrift*, No. 26, 1872) the history of a case of abortion from the gynæcological clynic of Prof. Spiegelberg, of Breslau, and one from the polyclinic of that city, as striking demonstrations of the efficiency of this method, which was first proposed and practiced by Prof. Spiegelberg.

It is an accepted principle of obstetrics, that the removal of a retained placenta, in delivery at full term, should be accomplished early, to prevent dangerous hemorrhage, and to accomplish this object with the least possible lesion to the womb. It would, however, be decidedly erroneous to extend this principle to cases of abortion, as in these neither the danger of putrescence of the placenta in utero, and respectively of putrid resorption, nor that of profuse hemorrhage, is as great as in deliveries at full term; while the forcible introduction of the hand through the often firmly-contracted os, into a very small cavity, frequently much reduced in capacity by the expulsion of the ovum, is, if at all possible, a much more serious act of interference than the same proceedure in the capacious and yielding womb after premature delivery, or delivery at full term. While retained placenta after abortion does not demand immediate active interference, it constitutes a situation calling for the most strict watchfulness and attention of the physician, as, with the exception of very rare cases, more or less hemorrhage will occur, sooner or later, no matter how well the patient may have been doing for several days.

The occurrence of the first hemorrhage furnishes the indication for the forcible removal of the placenta, and no means accomplish this object with such absolute certainty and speed, and so little inconvenience, as the sponge tent. It dilates the internal os mechanically, excites intense contractions, and operates as a hæmostatic by mechanical occlusion of the os uteri. One or two large sponge tents are generally sufficient; and if any one still fears hemorrhage during his absence, a tampon can easily be applied below the tents. In some cases, the expulsion of the placenta takes place so rapidly, that the sponge tents alone are fully sufficient to prevent hemorrhage; in other cases, it passes slowly through the lower uterine segment and the cervical canal; and if severe hemorrhage accompanies this stage, the bottom of the vagina must be closed by a tampon.

Hypodermic Injections of Fluid Extract of Ergot (Secale Cornutum) in Fibro-Myome of the Uterus.—Prof. Hildebrandt of Koenigsberg (*Berliner Klinische Wochenschrift*, No. 25, 1872), has lately treated uterine tumors of this kind with injections of morphia into the cellular tissue of the integuments of the abdomen, with unexpectedly favorable and noteworthy results. He injects, generally once a day, the contents of a full Prava syringe of a solution of extract of secale cornutum (one-fifth extr. sec. cornut., four-fifths glycerine and aquæ distil. mixed in equal parts.) He relates the history of nine cases. One very large tumor, extending above the navel, disappeared entirely; another one, touching the edge of the ribs of the right side, and filling the entire abdominal cavity, was reduced until its upper border did not reach the navel. Four others were also materially diminished in size. Two cases did not submit to treatment long enough to determine the final effect of the remedy, although actual measurement showed that the tumors were diminishing in size. In one case, the treatment with ergotin was inadmissible, because it produced symptoms of poisoning. The profuse hemorrhages and serous discharges, and also the dragging pains belonging to all these cases, yielded very readily to this treatment—a fact which makes Prof. Hildebrandt's method so much more valuable, as extreme anæmia, resulting from these symptoms, often endangers the life of the patient.

It is of practical importance to know—

1. That the punctures made at the lower portions of the abdomen are much more painful than those made about the navel.

2. That the punctures are inclined to bleed about the time of the menstrual period, and that, during the real existence of that discharge, the injections are generally omitted.

3. That after the injections have been made ten to fifteen times, the injected fluid frequently flows out again through the opening made by the needle, and that it becomes necessary to apply a pit of lint moistened with collodion over the aperture.

Acetic Acid an Effective Remedy in Psoriasis.—Dr. Burk, Physician to the Hospital at Lübeck, claims (*Berliner Klinische Wochenschrift*, No. 14, 1872) that etiological treatment, fulfilling only rational indications, is an impossibility in chronic diseases of the skin, particularly in psoriasis, and resorts to a persistent external alterative and destructive method for the cure of this disease, with decided success. After softening, loosening and removing, by the aid of warm soap-suds and a soft brush, the morbid epithelial scales over a portion of the surface, pure acetic acid is applied by the aid of a large camel-hair brush. This treatment, which causes a good deal of local pain, is continued as often, and extended over as large surfaces of diseased skin, as the endurance and energy of the patient will permit. The softened epithelial flakes dry up and fall off, or are easily removed before every new cauterization. Intense pain and diffuse œdema of the skin sometimes require the temporary suspension of this treatment, and call for the use of cold lotions, lead water, etc.; but as soon as these symptoms subside, the acid must be used again. The applications are generally made from once to twice daily—seldom oftener. Four to six weeks are generally necessary for a complete cure. The majority of patients becoming convinced of the efficacy of this treatment, bear its inconvenience very patiently.

The fear of exciting metastatis by this treatment has not been sustained by experience in a single case.

The cheapness, cleanliness and simplicity of this method, and the complete control which the physician can exercise as to the amount of surface to be placed under treatment, are not the least advantages of this method.

Dr. Burk recommends acetic acid as a local external remedy for the removal of thickened and indurated epithelial scales of the epidermis generally, and expects some benefit from it in the milder grades of ichthyosis after the continued use of warm baths and emollient ointments have softened the hard, horny scales and warty excrescences of that disease.

BACTERIA—THEIR RELATION TO SEPSIS AND CONTAGION. Dr. Ferd. Cohn (*Berliner Klinische Wochenschrift*, No. 16, 1872) expressed the results of his investigations before the Section of Natural Sciences of the Silesian Society for Home Culture, on February 14, 1872. He considers bacteria the instigators of septic decomposition of organic substances, as much so as the yeast fungus instigates the alcoholic fermentation of saccharine substances. They need no sugar for their formation, but any fluid which contains, besides ammonia and nitric acid, any one non-nitrogenous, carbonaceous substance, suffices for their production and multiplication: therefore, solutions of tartrate of ammonia, benzoate of ammonia, tartrate of potash with nitrate of ammonia, will develop bacteria; while tartrate of potassa, nitrate of ammonia, a solution of urea, will not develop them. Their office in the development of septic decomposition consists in a division of the albuminous organic compounds into ammonia, which they assimilate, and in other side products occurring in the process of decomposition. Finally, the speaker expressed the opinion that bacteria, which have been proven to exist in the human blood, and the secretions of the body in a number of contagious diseases in the form of ball bacteria, are very probably the bearers of infection, and that, by chemical division of the constituents of the blood, they produce a deleterious decomposition of that fluid. The drinking water, according to his investigations, must play a very important part in the transmission of these bodies.

New Method of Puncturing the Bladder in Retention from Enlarged Prostate.—In the *Medical Record*, July 1st, Dr. H. K. Clark, of Geneva, New-York, reports a case of enlarged prostate, in which Dr. George N. Dox and himself punctured the bladder several times within a few days, without inducing any unpleasant symptoms—the patient frequently dressing himself immediately after the operation, and walking about the house without any inconvenience. Dr. Clark contends that the results obtained in this case are of such a character as to demonstrate that, by his method, the operation of paracentesis cystitis is divested of its graver features, exhibiting the fact, that puncture of that viscus in retention, when other means fail, is almost entirely void of danger, and can be repeated *ad libitum*, without any serious consequences ensuing. Dr. Dox's operation is simply the puncture of the bladder just above the pubis, with a large-sized exploring trocar, and the removal of the canula as soon as the bladder is emptied—contending that if the canula is sufficiently small, there will be no danger of infiltration, and that frequently, in from twenty-four to thirty-six hours, all traces of the puncture of the skin will almost have entirely disappeared.

A Remedy Against Coryza.—Dr. E. Brandt, of Stettin, recommends (*Berlin Klin. Wochenscrift*, Nos. 12 and 18, 1872), as a remedy of remarkable efficacy in acute coryza and catarrhal affections of the respiratory tubes, the following formula, originally proposed by Dr. Hager (Pharmaceutische Centralhalle):

℞—Acid carbol. pul., grm. 5.0 (grs. 77.0.)
Spirit vini rectificat, grm. 15.0 (grs. 221.0.)
Liq. ammon. caust., grm. 5.0 (grs. 77.0.)
(Ponder specific 0.960.)
Aquæ distil, grm. 10.0 (grs. 154.0.)
Misce det at vitrum nigr. cum epistom vitreo.

He claims for it, most emphatically, a decided shortening of the first stage, abortion of the second, and amelioration of all the symptoms of coryza. It is to be used either by inhaling it frequently from the bottle, through the nostrils, as soon as possible after the commencement of the disease, or, still better, by inhaling a few drops from tissue paper, by holding it to the mouth and the nose in the hollow of the hand, and thus protecting the eyes from the effect of the remedy. Each inhalation is to be continued as long as the peculiar odor of the remedy is perceptible to the patient.

Tinctura Eucalypti Globuli—New Remedy Against Intermittent Fever.—Dr. Keller, Physician-in-Chief of the Austrian State Railway Company (*Berliner Klin. Wochenschrift*, No. 14, 1872), has caused this remedy to be extensively used by a number of the employés on the railways and the mines of Hungary and the Banat. The results are decidedly favorable, as a large number of cases, complicated with anæmia and enlargement of the liver and spleen, where quinine had failed, were cured by the use of this drug. This plant is a native of Australia, and has not, as far as we know, been introduced in the American market. It can be bought from Ch. Huber & Co., à Hyère (Var), near Toulon, France, at about three florins per pound; and as one pound will make two and a half pints of the tincture, one ounce would cost much less than five cents From two to three tea-spoonsful, given previous to the paroxysms, are said to be sufficient to arrest them. In very stubborn cases, larger doses are necessary, and in malarial cachexia, small doses must be used morning and night for some length of time.

Solid Food in Typhoid Fever.—Dr. Samuel D. Turner, of Circleville, Ohio (*The Clinic*), dissents from the views of Flint, Niemeyer, Reynolds, Murchison, and others, in regard to use of fluid food in typhoid fever. His experience of eighteen years has taught him that the advantages of solid over fluid food in this disease are as great as the latter is acknowledged to be over the older practice of semi-starvation. He has yet to see the case in which solid food has aggravated in any way a single symptom. Where this has been properly administered, he has never known death from perforation of or hemorrhage from the bowels, or from intractable diarrhœa.—*The Medical Record.*

Hypodermic Injections of Solution of Morphia in Melancholy.—Dr. Mendel, of Perkow, in a lecture delivered before the Medical Society of Berlin, March 20th, represents the mental depression existing in that disease as a hyperæsthesia of the brain, requiring for its removal, like any other hyperæsthetic part, absolute freedom from external exciting impressions, perfect quietude, and daily injections of solution of morphia—not, as cases of that kind are frequently treated, external excitement and change of impressions by traveling, visiting theatres and concerts, etc. He commences with one-eighth grain doses, morning and night, and increases rapidly to one-half, and even one grain, with decidedly beneficial effect.

Chloral Hydrate in Spasmus Glottidis.—(*Jarbuch für Kinderkrankheiten,* 4 H., 1871.—Dr. Rehn treated a case of spasmus glottidis, which had assumed a threatening character through the severity and frequency of the attacks, with chloral hydrate. The attacks were perceptibly lessened, and finally quieted. The child thus treated was seven months old. The medicine was well borne, and produced no disturbances of digestion.—*New-York Medical Journal.*

Mono-Bromate of Camphor in Delirium Tremens.—In the July number of the *New-York Medical Journal*, Dr. Allen McLane Hamilton reports a case of delirium tremens, in which is illustrated the advantages of mono-bromate of camphor in this terrible affection. After trying the bromide of sodium in large doses—at first alone, and later, with hyoscyamus and lupulin, without inducing sleep, he had a number of pills prepared after the following prescription: ℞—Mono-bromate of camphor, ʒj; confection of roses, q. s. Mix and divide in twelve pills.

One pill was given, and within a half hour, the patient fell asleep, and slept soundly for thirteen hours; seven hours later, he took another pill, and slept four hours. From this prescription, he obtained regular sleep, and awoke always bright and refreshed. No bad effects followed even when he took ten grains.

Traumatic Amaurosis Successfully Treated by Injections of Strychnia.—Dr. Werner, of Sangerhausen, Germany, has, in imitation of Prof. Naegel, of Tübingen (*Berliner Klinische Wochenschrift*, No. 6, 1871), used hypodermic injections of strychnia in a case of complete amaurosis of the left eye, caused by a wound of the lower eyelid and sclerotic membrane, inflicted by a small fragment of shell. Three injections of strychnia made in four days—the weakest containing 0.0002, the strongest 0.0008 grms. (one-thirtieth to one-eighth grs.)—established a prompt cure.

An Aged Operator and a Tough Patient.—Dr. Joseph Stevens (*The Clinic*), aged eighty-two years, recently amputated the thigh of a patient sixty-six years old, for obstinate and extensive ulceration of the foot and leg, of forty years' duration. On the second day, the patient sat up in bed and shaved himself, and before the end of a week, was able to get out of bed without assistance each morning, to have his bed arranged.—*The Medical Record.*

